# Fully covered self-expandable metal stent with an anti-migration system vs plastic stent for distal biliary obstruction caused by borderline resectable pancreatic cancer: A protocol for systematic review

**DOI:** 10.1097/MD.0000000000018718

**Published:** 2020-01-17

**Authors:** Takashi Tamura, Hiroki Yamaue, Masahiro Itonaga, Yuki Kawaji, Junya Nuta, Keiichi Hatamaru, Yasunobu Yamashita, Yuji Kitahata, Motoki Miyazawa, Seiko Hirono, Ken-ichi Okada, Manabu Kawai, Toshio Shimokawa, Masayuki Kitano

**Affiliations:** a Second Department of Internal Medicine; b Second Department of Surgery; c Clinical Study Support Center, Wakayama Medical University, Wakayama, Japan.

**Keywords:** biliary obstruction, borderline resectable pancreatic cancer, fully covered self-expandable metal stent, plastic stent

## Abstract

**Background and aim:**

Biliary obstruction can impair the effectiveness of neo-adjuvant chemotherapy. This study was designed to compare biliary stenting with covered self-expandable metal stents (FCSEMS) and plastic stents (PS) in patients with biliary obstruction caused by borderline resectable pancreatic cancer (BRPC) who were undergoing neo-adjuvant chemotherapy during preoperative biliary drainage.

**Methods:**

This single-center, comparative, randomized, superiority study was designed to compare FCSEMS with PS for drainage of biliary obstruction of BRPC. Twenty two eligible patients providing informed consent will be randomized 1:1 by computer to either FCSEMS or PS for endoscopic retrograde biliary drainage (ERBD). All subsequent clinical interventions, including crossover to alternative procedures, will be at the discretion of the treating physician based on standard clinical care. The primary outcomes will be the rates and causes of stent dysfunction during preoperative biliary drainage. Other outcomes include time required for ERBD, adverse events related to ERBD, period from ERBD to surgery, percentage of patients able to undergo surgery, operation time, intraoperative bleeding volume, postoperative adverse events, and postoperative hospitalization. Subjects, treating clinicians, and outcome assessors will not be blinded to assignment.

**Discussion:**

This study is intended to determine whether FCSEMS or PS is the better biliary stent for ERBD for management of patients with biliary obstruction of BRPC, a common clinical dilemma that has not yet been investigated in randomized trials.

**Trials registration:**

UMIN-CTR, Identifier: UMIN000030473. Registered July 10, 2017, Wakayama Medical University Hospital.

## 1 Introduction

The only curative treatment for pancreatic cancer is surgery. In many patients, however, pancreatic cancer is unresectable at diagnosis. Pancreatic cancer thus has one of the poorest prognoses among malignant tumors.

Neo-adjuvant chemotherapy (NAC) was recently introduced to reduce tumor size before surgery and to improve the radical resection (R0 resection) rate.^[1]^ Because tumor invasion of large vessels reduces tumor resectability, patients undergo NAC to reduce the invasion of large blood vessels, such as the celiac and superior mesenteric arteries. Current National Comprehensive Cancer Network (NCCN) guidelines distinguish pancreatic cancer with large blood vessel infiltration from tumors that can undergo R0 resection. The curative resection rate in patients with borderline resectable pancreatic cancer (BRPC) is significantly higher in those who do than do not receive NAC.^[1]^ NAC protocols, however, have not yet been standardized, although the optimal treatment period from the beginning of NAC to surgery ranges between 2 and 6 months.^[1]^ Interruptions of chemotherapy can lead to cholangitis, so patients with distal biliary obstruction caused by pancreatic cancer require biliary duct drainage until surgery. Endoscopic retrograde biliary drainage (ERBD) may be accomplished in patients with biliary obstruction of resectable pancreatic cancer by insertion of a plastic stent (PS). PS for biliary duct obstruction of the BRPC is most frequently inserted prior to NAC. However, NAC prolongs the time required for biliary drainage of the obstruction. Chemotherapy may therefore have to be discontinued due to cholangitis from PS dysfunction.^[2]^

An alternative to PS in patients with BRPC is the insertion of a self-expandable metal stents (SEMS), usually by ERBD. SEMS have a longer patency than PS, and covered SEMS have a significantly longer patency than uncovered SEMS.^[3]^ Covered SEMS is reported to be useful in patients with resectable pancreatic cancer and distal biliary duct obstruction.^[4]^ Moreover, several retrospective and prospective studies found that SEMS prior to NAC was useful in patients with BRPC and distal biliary obstruction.^[5–11]^ However, in many institutions, even now, PS is used for biliary drainage of distal biliary obstructions of patients with BRPC..^[8–11]^ However, to our knowledge, no prospective studies have compared the placement of PS and SEMS in patients with BRPC and biliary duct obstruction. PS has therefore been used in patients with BRPC, because there have been concerns about the influence of SEMS on surgery. The current randomized study aims to compare the efficacy during preoperative biliary drainage between fully covered SEMS (FCSEMS) for BRPC with biliary duct obstruction and PS for that.

## 2 Methods

### 2.1 Design

This is a prospective, single-center, randomized trial. The study protocol was approved by our institutional review board and performed according to the guidelines described in the Helsinki Declaration for biomedical research involving human subjects (Clinical trial registration number: UMIN ID000030473)

### 2.2 Patients

Informed consent will be obtained from all study subjects. Patients with an initial diagnosis of BRPC and malignant biliary obstruction will be evaluated. Patients will be included if they are >20 years old, have a malignant distal biliary obstruction, and have been diagnosed histologically or cytologically with pancreatic adenocarcinoma consistent with NCCN guidelines (version 2, 2016). Pathological tissue will be obtained by endoscopic ultrasound-guided fine needle aspiration prior to inclusion of patients in the study. Further inclusion criteria are BRPC with arterial and/or venous involvement; this will include patients who have an Eastern Cooperative Oncology Group (ECOG) performance status of 0–1 and are scheduled to undergo NAC. Both patients who underwent previous biliary drainage in the other hospitals and those without previous biliary drainage will be also included.

Patients will be excluded if they have severe dysfunction in other organs (American Society of Anesthesiologists physical status classification grade III or IV), are diagnosed with resectable or unresectable pancreatic carcinoma according to NCCN guidelines, and/or and have intestinal stenosis on the anal side.

### 2.3 Randomization and blinding

**Figure 1:**
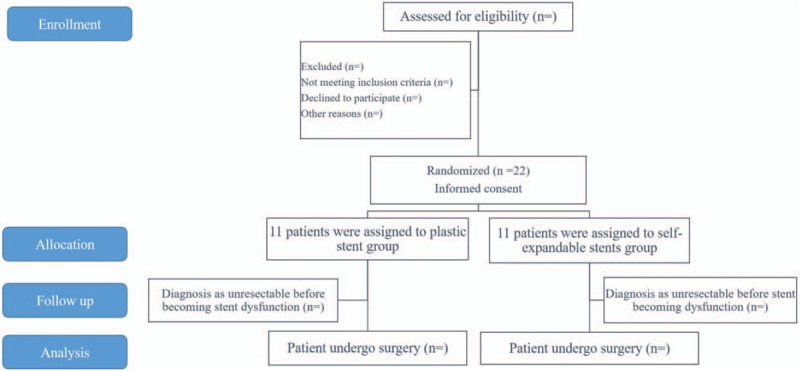
Protocol workflows for the fully covered self-expandable metal stent with an anti-migration system and the plastic stent, showing their comparative efficacy and safety for distal biliary obstruction caused by borderline resectable pancreatic cancer during preoperative biliary drainage.

Written consent will be obtained from patients with BRPC and obstructive jaundice who fulfill the eligibility criteria. These patients will be randomized 1:1 by the central registry of Wakayama Medical University to the FCSEMS group or the PS group as the first intervention. Study of each group will be carried out after a series of consecutive numbers have been assigned to the patients by the central registry of Wakayama Medical University (Fig. 1).

Although physicians cannot be fully blinded to stent use, because they have easy access to medical records and endoscopic images; those who place the stents will be requested not to reveal the type of stent used to those who decide when to intervene at the time of stent dysfunction. Data analysts will be blinded to group allocation (dummy coded) when conducting statistical analyses.

### 2.4 Data collection

**Figure 2:**
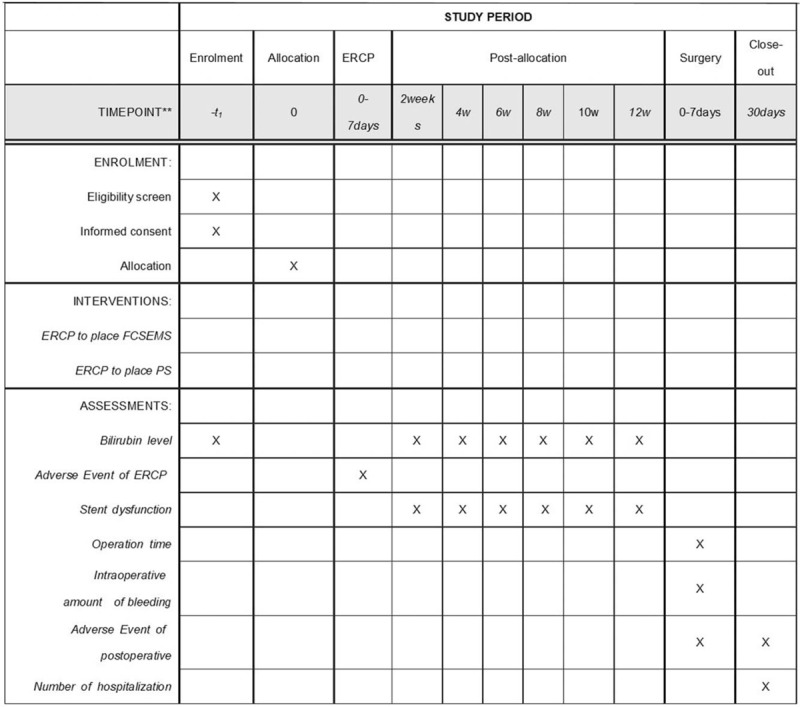
Trial schedule.

Data is collected prospectively for all patients including history, physical examination, laboratory data, pathologic examination, perioperative clinical information, and complications. The study allocation, interventions, and assessments are adapted from the Standard Protocol Items (Fig. 2).

### 2.5 Data monitoring and audit

Monitoring Committee will independently review the interim analysis reports and stop the trial early if necessary. Central monitoring will be performed each year by the data center to evaluate study progress and ensure study quality. The following aspects will be monitored:

1. data accumulation,
2. patient eligibility,
3. severe adverse events,
4. protocol deviations,
5. reasons for cessation or expiration of the protocol,
6. background factors of the patients, and
7. other problems concerning study progress and safety.

All study documentation and the source data/documents will be accessible to auditors/inspectors, and questions will be answered during inspections.

### 2.6 Procedures

**Figure 3:**
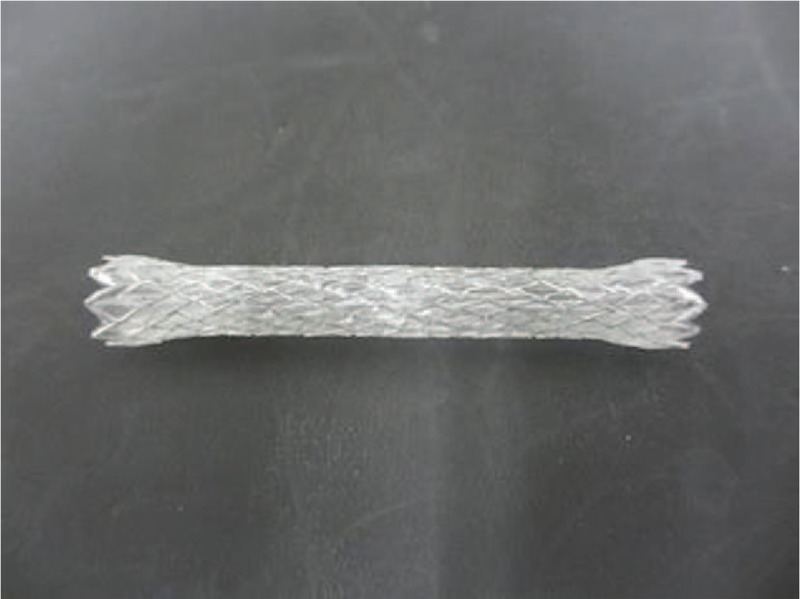
Photograph showing the fully covered self-expandable metal stent used in this study. A fully covered self-expandable metal stent with flares at both ends is composed of laser-cut platinum-cored nitinol. The stent is covered by 2 layers of film, a polyurethane inner layer and a silicone outer layer. The diameter of the stent is 10 mm and the diameter of the flare is 11.5 mm.

After sphincterotomy, a fully covered SEMS (bile rush fully covered metal stent, Piolax, Yokohama, Japan) or a 10 Fr PS (Zimmon Biliary Stent, Cook Endoscopy, Winston-Salem, NC, USA) will be inserted at the site of biliary duct obstruction during endoscopic retrograde cholangiopancreatography (ERCP). In patients who underwent previous biliary drainage in other hospitals, the same procedure will be performed after removal of the previous stent. Each fully covered laser-cut SEMS with flares at both ends is composed of a platinum-cored nitinol wire and covered by 2 layers of film, the inner layer made of polyurethane, and the outer layer made of silicone membrane. The diameters of the FCSEMS and flare are 10 mm and 11.5 mm, respectively (Fig. 3), with the length of the FCSEMS (60 mm or 80 mm) inserted depending on the location and length of the biliary stricture. The flare is intended to prevent stent migration, a potential adverse event of FCSEMS implantation. Each FCSEMS will be deployed to extend at least 1 cm above the top of the stricture and approximately 5 mm into the duodenum.

**Figure 4:**
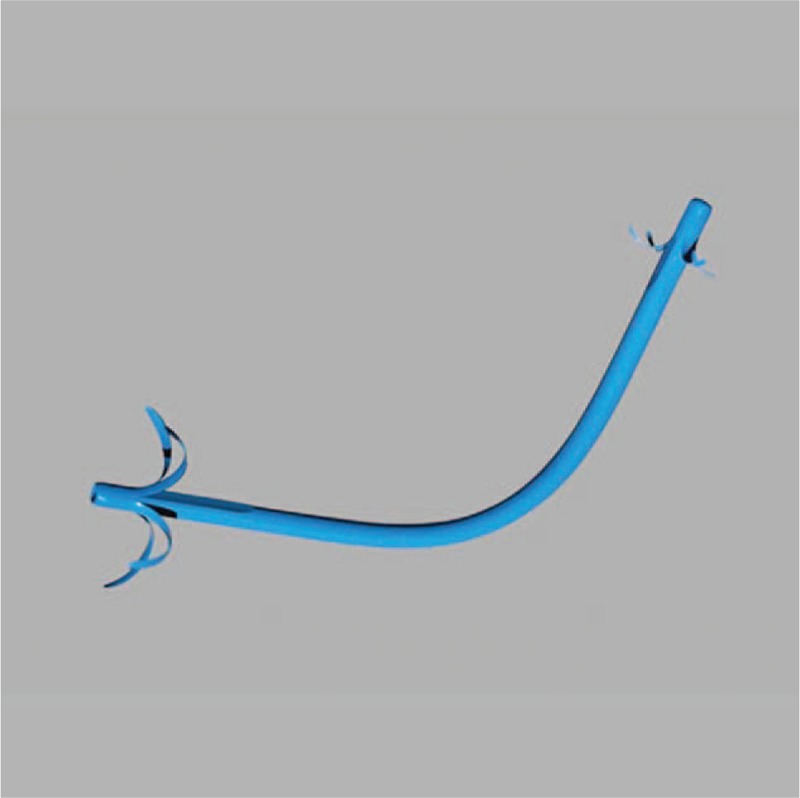
Plastic stent used in this study. The plastic stent is made of polyethylene. It has a diameter of 10 Fr and a length of 70 mm.

Each PS is composed of polyethylene and is 10 Fr in diameter and 70 mm in length (Fig. 4). None of the plastic stents used in this study are 60 mm or 80 mm in lengths. PS with length of 70-mm for biliary drainage is used.

### 2.7 Follow-up

Patients will be periodically followed-up after stent insertion at the same hospital. Improvement of jaundice will be determined by measuring serum total bilirubin concentration 2 and 2 weeks after stent deployment. Stent dysfunction and adverse events will be monitored every 2 weeks. Stent dysfunction is defined as a diagnosis of jaundice or cholangitis, as previously described.^[12]^ Patients who experience deterioration in condition, specifically jaundice with high-grade fever, will be evaluated at their local hospital and undergo interventions as outpatients. Otherwise, patients will be evaluated every 2 weeks as outpatients, with evaluations including physical examinations, complete blood cell counts, and blood biochemistry, including liver function tests. If any changes in liver function or findings related to inflammation are observed, patients will be evaluated by imaging modalities, including ultrasonography, computed tomography, and magnetic resonance imaging. If bile duct dilation is observed, re-intervention will not be performed until serum total bilirubin concentration is increased or the patient experiences acute cholangitis refractory to antibiotics. Data on chemotherapy will be collected after stent insertion. To evaluate the effects of surgery on stent insertion, operation time, postoperative complications, and intraoperative amount of bleeding will be recorded. Patients will be monitored daily for the first 30 days after surgery; and any postoperative adverse events will be recorded. Patients who do not undergo pancreatectomy will be censored when pancreatic adenocarcinoma is diagnosed as unresectable.

### 2.8 Outcomes

The primary endpoint will be the rate of stent dysfunction throughout the follow-up period. This parameter is defined as the rate of re-intervention (the number of patients in each group undergoing re-intervention/the number of patients in each group). Re-intervention will be indicated when serum total bilirubin concentration is >2 mg/dl or serum AST, ALT, ALP or γ-GTP concentration is ≥1.5 times the median standard value. If these concentrations are not completely normalized after ERCP, re-intervention will be performed when total bilirubin, ALP, γ-GTP, AST or ALT concentrations are higher than during the previous blood test.

Secondary endpoints will include:

1. the procedure time for ERCP, defined as the time from scope insertion to scope removal;
2. adverse events related to ERCP, defined according to standard consensus guidelines^[13]^;
3. the time (days) from ERCP to surgery;
4. the percentage of patients able to undergo the final surgery, defined as the percentage who undergo pancreatectomy surgeries except trial laparotomy; and
5. the total operation time.
6. intraoperative bleeding volume, defined as bleeding volume from laparotomy to closure;
7. postoperative adverse events, defined according to the Clavien-Dindo classification^[14]^; and
8. postoperative hospitalization, defined as the number of days from surgery to discharge.

However, in patients scheduled for postoperative chemotherapy during hospitalization, the chemotherapy start date is regarded as the day discharge is permitted, and the postoperative hospital stay is defined as the number of days from surgery to the start of chemotherapy.

### 2.9 Statistical analysis

The primary analysis will be a superiority comparison between FCSEMS and PS for the primary endpoint, rate of stent dysfunction throughout the follow-up period. The required sample size to achieve statistical relevance was determined based on a retrospective study, in which the stent dysfunction rates until surgery for FCSEMS and PS were 85.7% and 17.6%, respectively.^[7]^ To demonstrate a 68.1% difference in the stent dysfunction rate, using statistical power of 80% and assuming a one-sided error rate of 0.025, at least 20 randomly assigned patients will be required. Assuming a loss to follow-up of 10%, at least 22 patients will be required.

The full analysis set (FAS) will include all randomized subjects, except for those who cannot undergo stent deployment. The primary study endpoint, a comparison of the rate of stent dysfunction in the FCSEMS and PS groups, will be analyzed in the FAS using Fisher exact test. Patient characteristics and secondary endpoints, including complications of ERCP, period until start of NAC, period from the start of NAC to surgery, the percentage of patients able to undergo surgery, operation time, intraoperative amount of bleeding, postoperative complications, and period of postoperative hospitalization, will be analyzed in the FAS or the per-protocol sample (PPS) using odds ratio with 95% confidence intervals (CIs) or median differences with 95% CIs,^[15]^ as appropriate. The PPS was defined as all randomized subjects, except those diagnosed with unresectable pancreatic adenocarcinoma after allocation from the FAS.

## 3 Discussion

To our knowledge, the trial is the only current randomized comparison of the efficacy and safety of FCSEMS and PS for BRPC in patients with distal biliary obstruction. This condition has remained difficult to address because of its associated practical and methodological complexity.

For practicality and generalizability, we adopted a pragmatic study design that includes only 1 protocol-driven interventional randomization, for either PS or FCSEMS. All other clinical decisions are at the discretion of the treating physician, as clinically appropriate.

A retrospective study reported that FCSEMS is more effective than PS for biliary obstruction of BRPC.^[5–7]^ Another retrospective study involving 79 patients scheduled to undergo pancreatectomy for pancreatic cancer with biliary obstruction following NAC found that the frequency of cholangitis was significantly lower after implantation of metal stents than PS.^[5]^ Placement of metal stents is thus useful in allowing NAC for patients with biliary obstruction planning to undergo pancreatectomy. Furthermore, surgical and postoperative complication rates were similar following FCSEMS and PS implantation.^[5]^ In another study, 71 patients underwent preoperative SEMS placement, but none experienced adverse events.^[6]^ A retrospective study from Japan found that SEMS was useful for patients with resectable pancreatic cancer and biliary duct obstruction scheduled to undergo NAC.^[7]^ Previous studies have reported differences between the PS stent and FCSEMS in terms of time of placement, type of neo-adjuvant chemotherapy, the presence of a tumor, and selective bias by the endoscopist.^[5–7]^ In addition, because BRPC has a tumor size that is often smaller than that of unresectable pancreatic tumors, the tumor may be reduced by neo-adjuvant chemotherapy and FCSEMS may cause more migration than in previous reports.^[16,17]^ It may be difficult to resolve stent dysfunction caused by FCSEMS migration. On the other hand, PS stents are easy to exchange, even when stent dysfunction has occurred. Regarding the site of placement, the site of placement of PS has little influence on surgery. However, surgery may be influenced if FCSEMS is placed too close to the hilum. The safety of SEMS, including concerns over its potential influence on surgery, has not been proven to be the same as that of PS. This randomized control trial in a single institution may eliminate bias of surgery and NAC, a bias not eliminating in retrospective studies. Therefore, we have planned a clinical study to examine the efficacy and safety of FCSEMS placement for biliary obstruction of BRPC with NAC. This study assesses the use of a newly designed FCSEMS, with flares at both ends. The large flared ends may prevent stent migration, a drawback of FCSEMS.^[16]^ We used 10 Fr PS, a wider plastic stent than that used for ERBD, to prevent stent dysfunction during NAC. Results showing that FCSEMS is more effective than PS for biliary obstruction of BRPC, and that the safety of the 2 types of stent is similar, suggest that FCSEMS may be selected for biliary obstruction at diagnosis of BRPC. Due to the progression of pancreatic cancer during NAC, BRPC may be diagnosed as unresectable. Insertion of an FCSEMS, which has a long duration of patency in unresectable pancreatic cancer, may reduce the frequency of biliary stent obstruction and hospitalization.

This study will be the first to determine whether FCSEMS or PS is the better stent for biliary obstruction of BRPC prior to NAC.

## Acknowledgments

We acknowledge proofreading by Benjamin Phillis, Clinical Study Support Center, Wakayama Medical University.

## Author contributions

**Formal analysis:** Toshio Shimokawa.

**Investigation:** Hiroki Yamaue, Masahiro Itonaga, Yuki Kawaji, Junya Nuta, Keiichi Hatamaru, Yasunobu Yamashita, Yuji Kitahata, Motoki Miyazawa, Seiko Hirono, Ken-ichi Okada, Manabu Kawai.

**Writing – original draft:** Takashi Tamura.

**Writing – review & editing:** Masayuki Kitano.
